# Spatial Assessment of Land Degradation Risk in the Choke Mountain Watershed

**DOI:** 10.1155/tswj/6657991

**Published:** 2026-04-25

**Authors:** Alelgn Ewunetu

**Affiliations:** ^1^ Department of Geography and Environmental Studies, Woldia University, Woldia, Ethiopia, wldu.edu.et

**Keywords:** Choke Mountain upper Blue Nile, degradation indicator, GIS, MCA, watershed

## Abstract

Assessing land degradation is essential for identifying susceptible regions and planning sustainable landscape management approaches. This research employed a combination of geographic information system (GIS) and multicriteria analysis (MCA) to delineate and evaluate land degradation within the Choke Mountain watershed of the upper Blue Nile. The Analytical Hierarchy Process (AHP) was employed to standardize all indicators and assign weights through comparison. A comprehensive analysis of physical, chemical, and biological indicators of land degradation was carried out. The results showed that about 50.64% of the watershed is at a high to very high risk of soil erosion, with an average loss of 44 t of soil per hectare each year. More than half of the watershed also exhibits moderate‐to‐high biological degradation levels, as evidenced by sparse vegetation cover and low levels of soil organic matter. About 70.7% of the area experiences only a mild physical degradation type. Biological degradation was rated as low in 37.4% of the watershed and moderate in 55.5%. The chemical degradation assessment revealed that most of the area (55.6%) has neutral soil pH values between 6.7 and 7.3. The integrated MCA results showed that 1.2% of the watershed is very low, 25.5% is low, 37.15% is moderate, and 36.15% is highly degraded in the Choke Mountain watershed. Overall, the main causes of land degradation in the Choke Mountain watershed are severe soil erosion, deforestation, and biomass deterioration. The most evident signs of land degradation are extensive biodiversity decline and soil erosion. Therefore, implementing comprehensive land management strategies is essential to prevent land degradation, enhance soil organic matter, and increase vegetation cover.

## 1. Introduction

Land degradation is expressed in the persistent reduction or loss of land resources′ value to provide ecosystem services [1, 2]. It reduces the land′s capacity to provide essential ecosystem services, including biodiversity conservation, food production, water regulation, and climate regulation [1–3]. According to the UNCCD (2015), about 24% of the Earth′s land surface is affected, which consists of about 10% of grasslands, 20% of cropland, and 30% of forests [3]. Africa is especially at risk because about 30% of its productive land has been degraded, which poses serious risks to food security and agriculture [4]. Land degradation happens through several biophysical processes, including the loss of essential soil nutrients, soil erosion, soil acidification, and a decrease in the amount of water available [5].

Land degradation in Ethiopia has been a major problem that affects a large percentage of land ([6–9]), with well over 85% of the land experiencing it [10]. Some of the major causes include a growing population, poverty, a rough landscape, climate change, the heavy consumption of biomass fuels, and unsustainable farming practices [7, 9, 11]. These factors result in deforestation, soil erosion, biodiversity loss, and changes in hydrological patterns [12, 13]. In the early 20th century, the percentage of land covered by forests was more than 40%.Then in 2016, the percentage of land covered by forests was 12%. However, in 2023, the percentage of land covered by forests has increased to 23.6% [14, 15]. Recently, estimates suggest the forest cover increased again to 23.6% by 2023 [16]. This has been a result of the Green Legacy Initiative (GLI), which was established in 2019, leading to the planting of billions of saplings in the country in tree‐planting campaigns every year [17]. Soil erosion is still an issue in the highland regions of Ethiopia [13]. According to various research findings, it has been identified that in the northern highland regions, there is an annual loss of topsoil measuring approximately 45 t per hectare [18], whereas in northwestern regions, it is approximately 32.84 t per hectare [19]. In addition, soil acidity is also becoming an issue in the highland regions [5, 20].

The watersheds of Choke Mountain are located in the northwest highlands of Ethiopia, which have been considered an ideal place for farming activities. However, in recent times, this place is witnessing rapid land degradation, fragmentation of ecosystems, and a decline in land productivity ([21]; [22]. This watershed is considered a vital place for scientific investigation because it is the headwaters of a vital Nile tributary [22–24].

Some studies have been conducted on the biophysical changes in the upper Blue Nile basin using remote sensing and GIS approaches. However, most existing studies remain fragmented, typically focusing on a single dimension such as soil erosion, vegetation loss, or soil acidity [23, 25–28]. This is a major limitation of the studies conducted so far on the biophysical changes in the Blue Nile basin [5, 29], in that they overlook the multidimensional nature of the changes and the lack of comprehensive indicators that integrate the chemical, biological, and physical attributes of the land resources. They also overlook the decision‐support approaches that require reproducible and integrative approaches.

In order to fill these gaps, the present study employed an inclusive approach based on the Revised Universal Soil Loss Equation (RUSLE), multicriteria analysis (MCA), and the Analytical Hierarchy Process (AHP) in a GIS environment. This study represents the first instance in which the above approach was used to evaluate land degradation in the watershed area of Choke Mountain [23]. This study also represents one of the few attempts to apply the above approach in the highlands of Ethiopia [7, 9, 29]. However, continuous monitoring of land and water resources is also necessary due to dynamic changes in nature and human activities [30]. Land vulnerability mapping can also provide support in taking effective actions to address the problem [24, 31].

The research combines robust methodology and context to provide a comprehensive overview of land degradation. By using RUSLE, GIS, MCA, and AHP, this research extends conventional one‐dimensional research by employing a robust multi‐indicator approach that is based on a comprehensive evaluation [5]. Simultaneously, this research has enabled the evaluation of chemical, biological, and physical factors. It has provided a clear and reproducible index of land degradation. In this research, a multidimensional evaluation of Choke Mountain has been conducted. Choke Mountain has traditionally been considered a region of high agricultural productivity; however, it is currently experiencing considerable pressure from climate and land degradation [21, 22]. This research is helpful for environmental policy and sustainable land management and has provided a comprehensive overview of this region. It has also provided a comprehensive framework that can be used for other regions of the highlands of Ethiopia.

Therefore, the purpose of the study is to assess the land degradation vulnerability in the Choke Mountain watershed of the upper Blue Nile basin by combining GIS and MCA techniques. Specifically, the objectives of the study are as follows: (1) to quantify the amount of soil loss in the watershed annually, (2) to create a map that highlights the major biological, physical, and chemical land degradation indicators, and (3) to create a map that presents the land degradation index in the watershed comprehensively.

## 2. Methods and Materials

### 2.1. Description of the Study Area

**Figure 1:**
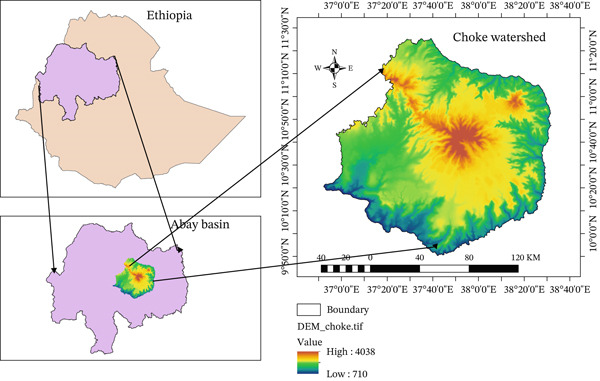
Map of the Choke Mountain watershed.

The Choke Mountain watershed is located in the highlands of Ethiopia, in the upper Blue Nile region, at 10.8°–11.9°N and 38.2°–39.6°E. It is a vital contributor to the Blue Nile (Abay) River basin, covering 1,994,620 ha in total. Choke Mountain is a water tower for the upper Blue Nile basin and the birthplace of over 60 rivers and 270 springs, giving it the reputation of a biodiversity hotspot. The Choke watershed has a diverse range of slope and soil types, including tropical alpine environments at 710–4038 m above sea level, as well as the hot and arid environment of the Blue Nile gorge below 1000 m, including areas below 1000 m [22]. It is the birthplace of many rivers in the Nile basin and has six different agroecology zones with high biodiversity levels. The river water flows into the Blue Nile River and originates from the mountain and gorge environments that cover most of the region. Since Choke Mountain is not just a landmark but also provides more than 10% of the Nile′s water, thereby creating an ecosystem that favors Ethiopia and others at large (see Figure 1). Simane et al. [21] explain that the region comprises a broad agroecological zone that ranges from cold to very cold and moist sub–Afroalpine regions, moving to Afroalpine highlands, and finally from tepid to cool and moist mid‐highlands. According to the Ethiopian National Meteorological Agency, the region has a mean temperature of 19.4°C annually, with a range of 24.6°C–28.1°C and 11.0°C–14.5°C for maximum and minimum temperatures, respectively [21]. Rainfall in the region follows the ITCZ seasonal migration, resulting in most of the rainfall occurring during the summer season, that is, June to September [21].

**Figure 2:**
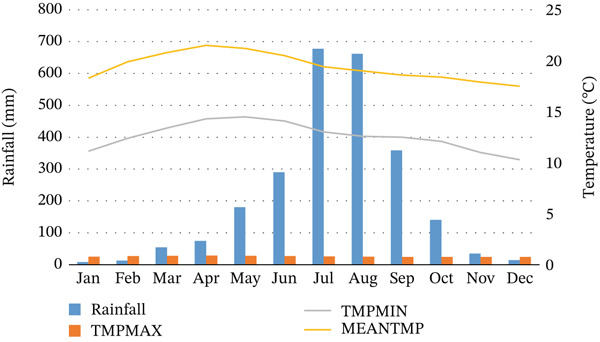
Average monthly rainfall and temperature records (1986–2022).

Meteorological records obtained from meteorological stations within the watershed and nearby areas indicate that the region receives an average annual rainfall of 1334.48 mm between 1986 and 2012, ranging from 810 to 1815 mm per annum (see Figure 2). The region has various types of soils, mainly nitisols, alisols, cambisols, leptosols, vertisols, and luvisols [22, 32]. In addition, the region is underlain by various geological formations, including basaltic rocks, where sandstone is dominant in the lowland areas [32]. The region has little native forest cover, mainly along river courses, slopes, and church grounds. In contrast, *Eucalyptus globulus* plantations cover the region, especially in the highland areas of the region, where the species is dominant as an introduced species.

The population living around Choke Mountain mainly depends on rain‐fed farming systems. This is highly susceptible to weather changes. The livestock raised around this area include cattle, oxen, sheep, and horses. One of the interesting aspects of farming around this region is that they still make use of the traditional Ethiopian Ard plow. This is also known as the Maresha plow. Although this plow is quite effective in loosening hard and dry tropical clay soils, it results in a huge amount of erosion. This is because they plow repeatedly to a certain depth. This results in a plow pan that restricts water infiltration and root growth [21, 22, 24]. In this watershed, overuse of grazing lands and deforestation is also causing problems. This is because both of these activities cause erosion. Moreover, with the increased demand for fodder and wood, natural resources are being exploited. This is causing problems for the environment around Choke Mountain [33].

### 2.2. Materials and Methods

#### 2.2.1. Nature and Data Sources

Landsat 8 OLI imagery acquired in January 2022 was obtained from the USGS Earth Explorer database. Similarly, elevation data were derived from the ASTER Global Digital Elevation Model (GDEM) (http://glovis.usgs.gov). Satellite‐derived precipitation datasets have become increasingly reliable alternatives to sparse rain‐gauge observations, particularly in data‐deficient areas like Africa, where in situ monitoring frequently falls short. The Climate Hazards Group InfraRed Precipitation with Station data (CHIRPS) is one of the most widely used products. It is used a lot in hydrological and environmental research and has been tested in Ethiopia [34] and has undergone validation under Ethiopian conditions [35]. Accordingly, CHIRPS rainfall records covering the period 2002–2022 were accessed from the (https://www.chc.ucsb.edu/data/chirps/) data portal. Thus, CHIRPS rainfall records for the years 2002–2022 were obtained from the CHC data portal. We also got soil property data from the African Soil Information Service (AfSIS) database with a spatial resolution of 250 m [36].

I collected 5500 ground‐truth points to help me test the accuracy of land use and land cover (LULC) classification. Field observations were performed in situ utilizing a handheld Garmin GPS, supplemented by georeferenced historical imagery from Google Earth Pro. The reference points were checked by going to the field and using the Google Earth time‐series tool. About 70% of the total samples were used to train the Random Forest (RF) classifier, and the other 30% were used to check its accuracy. Furthermore, qualitative insights were garnered from interviews with 10 elderly key informants to enhance interpretation and corroborate the quantitative findings.

### 2.3. Ethical Considerations

All methods were carried out in compliance with applicable guidelines and regulations. The Department of Geography and Environmental Studies at Woldia University gave this study ethical approval. A confirmation letter is included as Supporting Information. The department gave formal permission for the collection of data. Before taking part, they got informed consent. However, because the data collection happened after a fight between the Federal Government and local rebel groups, participants were worried about what might happen politically and did not want to give written consent. As a result, participants gave their verbal consent after being fully informed about the study′s purpose, how the data would be used, how their privacy would be protected, and their right to leave at any time without any consequences. No participants in the study were under the age of 18. All of the information that was collected was kept completely private. Identification information was only recorded for verification purposes and was kept safe until transcription and analysis were finished. I also got verbal permission to publish, and I made sure that the findings were reported in a way that kept the people who took part anonymous by putting them all together and summarizing them.

### 2.4. Spatial MCA Approach

**Figure 3:**
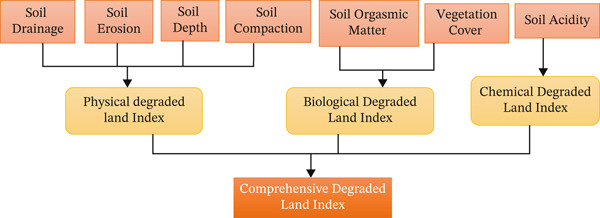
Methodological framework of the study (Source: Ewnunetu 2011).

Assessing land degradation requires integrating multiple indicators into a composite index [37]. MCA offers a good way to combine different indicators and rank decision options according to specific criteria [38]. The process typically includes setting goals, finding options, standardizing evaluation criteria, giving weights, using mathematical models, and putting the results together into a single index [5]. This study employed a spatial MCA integrated with the AHP to evaluate the severity of land degradation (Figure 3). The method was chosen because it could combine biophysical, climatic, chemical, and topographical variables into one index that could be compared (Figure 3). Using pairwise comparisons to calculate indicator weights made it possible to systematically prioritize and made the results stronger [39]. The MCA‐AHP framework is a good way to combine different geospatial datasets to solve difficult environmental problems when used with GIS [40].

To make all the criteria fit on a single scale from 1 (*very low*) to 5 (*very high*) degradation severity, the study used a linear scale transformation, which is one of the most common ways to standardize data in spatial MCA [41]. The pairwise comparison method was chosen because it has a strong theoretical basis and has been widely tested ([42; 43]). It is one of the three most common weighting methods. The study used the AHP framework to make raster layers for each indicator. Then, we did pairwise comparisons to figure out the weights. Less important criteria got the opposite value of more important ones, as shown in Figure 3. We used the principal eigenvalue and normalized eigenvector of the comparison matrix to figure out the weights for the criteria and subcriteria [5, 40]. Using the AHP framework, raster layers for each indicator were created, and pairwise comparisons were performed to determine weights, with less influential criteria assigned the reciprocal value of more influential ones, as shown in Figure 3. The weights for criteria and subcriteria were calculated using the comparison matrix′s principal eigenvalue and normalized eigenvector [40]. We checked the matrix for consistency and fixed any problems. After that, a weighted overlay procedure was used to combine the standardized criteria layers. All of the analyses were done in ArcGIS 10.5.

#### 2.4.1. Indicators of Physical Land Degradation

Physical degradation of the land is the impairment of the structure and the structural properties of the soil, such as texture, porosity, and the infiltration and moisture‐retention capacity of the soil [7, 9, 29]. In the present study, the physical degradation index is composed of four major indicators: soil erosion, soil compaction, drainage condition, and soil depth.

##### 2.4.1.1. Soil Erosion Factors and the RUSLE Model.

Soil erosion is a well‐known sign of land degradation [44]. In the Ethiopian highlands, especially in the Choke Mountain watershed, steep terrain and long‐term pressure on land use have made water‐driven erosion the main way that land is degraded [45]. The RUSLE is still a common way to figure out how much soil is lost each year on average. When used with GIS, it gives a useful framework for watershed‐scale assessment, especially in data‐scarce areas [46]. RUSLE does not account for gully or mass‐movement erosion processes [47]; however, its simplicity and compatibility with spatial analysis render it appropriate for this study [48]. Soil loss was calculated using Hurni′s [49] model parameterization.

(1) A=R×K×LS×C×P

where A is the estimated annual soil loss (t ha^−1^ yr^−1^); R is the rainfall erosivity factor (MJ mm ha^−1^ h^−1^ yr^−1^); K is the soil erodibility factor (t ha^−1^ MJ^−1^ mm^−1^); SL is the slope length and steepness factor (unitless); C is the land cover management factor (unitless); and P is the conservation practice factor (unitless).

##### 2.4.1.2. The Rainfall Erosivity Factor (R‐factor).

This shows how much rain can erode soil and move soil particles. The intensity, duration, drop size, and runoff energy of the rain all have an effect on it [46]. Because there were no automatic records of rainfall intensity in the study area, it was not possible to directly calculate rainfall energy. Consequently, mean annual rainfall data (2002–2022) from the CHIRPS dataset were utilized. The R‐factor was calculated using Hurni′s [49] empirical relationship, which has been used a lot in the Ethiopian highlands.

(2) R=−8.120.562+Pa

where “*R*” refers to rainfall erosivity (MJ mm ha^−1^ h^−1^ yr^−1^) and “*P* *a*” refers to the average yearly rainfall (mm).

##### 2.4.1.3. The Soil Erodibility Factor (K‐factor).

This measures how easily topsoil and surface materials can be washed away and moved by rain and runoff [50]. The main factors that affect it are the soil′s texture, permeability, organic matter content, structure, and overall stability [51]. Researchers have come up with different empirical formulas to guess the K‐factor for different areas [52]. In this study, we used the equation suggested by Ganasri and Ramesh [53], which has been used a lot in areas with different types of landscapes.

(3) K=0.20.3+exp0.0256SAN1.0−SIT100SITCLY+SIT0.31.0−0.25CC+exp3.722.95−C1.0−0.71100−SAN/1100−SAN/+exp−5.5122.9+1100−SAN/

where “*S* *I* *T*” represents silt in %; “*C*” refers to organic carbon in %; “*S* *A* *N*” is the percentage of sand; and “*C* *L* *Y*” is the percentage of clay.

##### 2.4.1.4. The Topographic Factor (LS‐factor).

This measures how the terrain affects soil erosion by combining slope length (L) and slope steepness (S) into one number [53]. Soil loss is greatly affected by both slope length and gradient. This is because surface runoff volume and speed go up when slopes are steeper and longer [25, 47]. The LS‐factor map in this study was created using these following equations:

(4) L=λ22.13m

(5) m=F1+F

(6) F=sinβ/0.8963.00.56sin β0.8+

In this context, **λ** represents the product of flow accumulation and cell size; L is the slope length factor; m is the slope length exponent; F is calculated assuming the soil is moderately susceptible to both rill and inter‐rill erosion; and *β* is the slope angle in degrees (converted to radians as slope in degrees × 0.01745).

(7) S=16.80.059∗sinβ− δ≥%

S=10.80.039∗sinβ+ δ<%

##### 2.4.1.5. The Cover Management Factor (C‐factor).

This shows how plants, crop cover, or conservation practices can help keep topsoil from being pulled away and moved [28]. The LULC map (Figure S4) that was used to get the C‐factor in this study was made from Landsat 8 images from January 2022 using supervised classification in ArcGIS. To give each LULC category a C‐factor value based on what the literature says (Table S1), the classified raster map was changed to vector format. ArcGIS 10.5 then turned the vector map back into a raster layer so that it could be added to the RUSLE model.

##### 2.4.1.6. The Support Practice Factor (P‐factor).

This tells how well terracing, contour farming, and strip cropping work to keep soil from washing away [5, 45]. Field observations indicated that terraces are extensively utilized within the watershed; however, their effectiveness was constrained due to inadequate maintenance. Because of this, it was thought that directly measuring terraces would not be a good way to find *p* values. Instead, the P‐factor was calculated using a different method that combines LULC and DEM data, based on the work of Wischmeier and Smith [46]. The watershed was divided into agricultural and nonagricultural land. Agricultural land was then divided into six slope classes. A specific *p* value was given to each slope class, and a default value of 1 was given to all types of land use that were not agricultural (Table S2).

##### 2.4.1.7. Topsoil Loss.

**Figure 4:**
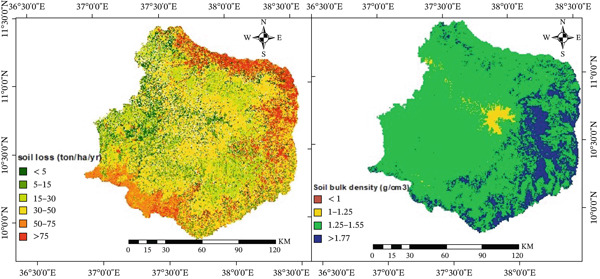
The annual soil loss (right) and soil bulk density (left) of the Choke watershed.

Topsoil loss in the Choke Mountain watershed was estimated by multiplying the five RUSLE factors, each resampled to a 30 × 30‐m spatial resolution (Figure S1, Equation (1)). The soil‐loss raster was divided into six levels of severity so that researchers could study the spatial distribution of erosion [5, 23, 25]. Model validation and reliability were evaluated by comparing them with empirical studies conducted in the Ethiopian highlands and through field verification. To check the predicted patterns of erosion in the field, the color‐coded topsoil loss map (Figure 4) was printed out and used on site.

##### 2.4.1.8. Soil Drainage.

Soil drainage refers to the movement and speed of water through the soil profile, including lateral flow and surface runoff. It has a big effect on soil morphology and related processes like waterlogging and crust formation [54]. The USDA [55] says that drainage is usually divided into seven groups based on how quickly water can be removed (Table S3). For this research, the soil drainage raster acquired from AFSIS was reclassified based on the categories and values delineated in Table S3.

##### 2.4.1.9. Soil Compaction.

Soil compaction is an important sign of physical land degradation [56]. It is the weight of oven‐dried soil per unit volume of topsoil and happens when pressure is put on the surface of the soil [57]. Soil bulk density is a common way to measure how compacted the soil is [58]. This study reclassified the soil bulk density raster from AFSIS based on the value ranges in Table S4. This made it possible to measure the levels of compaction across the Choke Mountain watershed.

##### 2.4.1.10. Soil Depth.

Soil depth is an important measure of physical soil quality [59, 55]. Soils with limited depth are typically more degraded and impose restrictions on root penetration and growth. In contrast, deeper soils better support plant development, as they usually retain greater amounts of nutrients and moisture [57]. To measure how the soil compacted was in the Choke Mountain watershed, this study used a soil bulk density raster from AFSIS and reclassified it based on the ranges shown in Table S5.

#### 2.4.2. Indicators of Biological Land Degradation

Biological land quality can be evaluated using a range of indicators. Commonly, soil organisms, vegetation cover, and topsoil organic matter are used to construct a biological land quality index [60], which is often made up of soil organisms, plant cover, and organic matter in the topsoil. This study utilized vegetation cover and soil organic matter (SOM) as primary indicators to delineate and quantify regions undergoing biological degradation.

##### 2.4.2.1. SOM.

SOM is an important part of soil that helps keep the quality of biological land and the stability of the whole ecosystem [31]. SOM is usually measured in a lab, but in this study, it was not possible to take samples directly at the watershed scale because of money and time constraints. Because (soil organic carbon) SOC makes up about 58% of SOM by weight, we used an AFSIS SOC raster as a stand‐in. Using Equation (8) in the Map Algebra raster calculator, we calculated SOM from the SOC raster, following the method of Combs et al. [61]. The resulting SOM raster was then reclassified using a scientific classification scheme (Table S6) to make it easier to map areas that have been biologically degraded.

(8) Percentage of organic matter=Percentage of total organic carbon×1.72

##### 2.4.2.2. Vegetation Cover.

Vegatation cover is an important sign of biologically degraded land because it shows how many plants there are and how much biomass they produce [5]. The soil‐adjusted vegetation index (SAVI) is often used in remote sensing to look at plants in places with a lot of exposed soil, where NDVI might not be accurate because of how bright the soil is [62]. This study computed SAVI from Landsat 8 imagery employing the calibration method delineated by Ewunetu and Kerbsh [5]:

(9) SAVI=NIR−REDNIR+RED+L1+L

where RED and NIR stand for reflectance in the red and near‐infrared bands, respectively, and L is a correction factor for canopy density that goes from 0 (*very dense vegetation*) to 1 (*very sparse vegetation*). Following Qi et al. [60], various L values (0.25, 0.5, and 0.75) were evaluated, and *L* = 0.5 was chosen based on visual analysis of the imagery and insights from prior research [26], indicating a moderate vegetation density appropriate for watershed‐scale analysis.

#### 2.4.3. Indicators for Chemical Degradation

##### 2.4.3.1. Soil Chemical Degradation.

Soil chemical degradation is when the chemical properties of soil change in ways that were not planned because the overall quality of the soil goes down [63]. Acidity, salinity, and sodicity are all common signs of healthy soil [64]. This study concentrated on soil acidity due to the humid conditions of the study area and the prevalent application of chemical fertilizers in conjunction with the removal of crop residues [5]. Soil pH was used to measure soil acidity. This is because soil pH shows how many hydrogen ions (H^+^) are in the soil solution [64]. I got a soil pH raster map from AFSIS and used a known soil pH classification scheme (Table S7) to reclassify it. This helped us see how acidic the soil was in different parts of the study area.

## 3. Results and Discussion

### 3.1. Indicators of Physical Land Degradation

#### 3.1.1. Estimation of Annual Soil Loss Using the RUSLE Model

The RUSLE model (Equation (1) and Figure S1) was used to estimate soil erosion in the study area. This model accounts for rainfall erosivity, soil erodibility, topography, land use/cover, and conservation practices to predict the amount of soil lost each year [23, 25]. The result from Equation (2) shows that the rainfall erosivity factor (R‐factor), derived from precipitation inputs, ranged from 610 to 1216 MJ mm ha^−1^ h^−1^ yr^−1^ (Figure S2). This high range corresponds to the erosive rainfall patterns characteristic of the Ethiopian highlands and intensively cultivated mountainous areas, where convective storm events exacerbate soil detachment [23, 25, 45].

SOC, texture, and structure were used to estimate the soil erodibility factor (K‐factor) in the Choke Mountain watershed Equation (3). The K‐values in the Choke Mountain watershed ranged from 0.075 to 0.375 t ha^−1^ MJ^−1^ mm^−1^ (Figure S2). Higher values were found in fine‐textured, low‐organic matter soils that are more likely to detach [53]. Earlier hydropedological studies in Ethiopian agricultural landscapes have revealed that soil texture and organic carbon utilize comparable effects on soil erodibility, mainly in contexts where LULC changes increase surface vulnerability [65, 66].

Topographic factors (LS) were calculated by multiplying slope length by slope steepness, resulting in LS‐values ranging from 1 to 109 throughout the watershed (Figure S3). Higher LS values were found mostly along steep midslope and lower slope corridors, which means there was a higher chance of overland flow concentration and sediment transport [45]. Similar terrain effects on soil erosion have been observed on Ethiopian escarpments, where steep gradients enhance uplift and shear forces [67]. The cover management factor (C‐factor) for this study was derived from the LULC map (Figure S4; Table S1), which achieved an overall classification accuracy of 90% and a kappa coefficient of 0.87. C‐factor values in the Choke Mountain ranged from 0.01 in forested areas to 0.6 in barren land (Figure S4). Lower C‐factor values, such as 0.01, indicate dense vegetation cover and, as a result, lower vulnerability to soil erosion, whereas higher values parallel sparsely vegetated areas that are more prone to erosion [5, 68]. Similar trends have been reported for tropical highland soils, where vegetation cover significantly reduces erosion risk [68]. Support practice factor (P‐factor) values ranged from 0.1 to 1 (Figure S5), with the highest values in lower and midwatershed segments dominated by grazing lands and minimal conservation measures. This gradient reflects the removal of protective vegetative cover and concentrated soil reinforcement, patterns corroborated in Ethiopian subcatchments under communal grazing and agricultural use [5, 45].

The RUSLE grids that were put together made it possible to make a 30 × 30‐m soil loss surface (Figure 4). The results show that the central watershed lost a moderate to high amount of soil each year (15–75 t ha^−1^ yr^−1^), whereas large areas of the steep upper and lower slopes lost more than 75 t ha^−1^ yr^−1^ (Table 1). About 35.9% of the watershed is in high to severe loss classes, which is due to the steep terrain, heavy rainfall, and lack of cover (Figure 4). These results are consistent with previous RUSLE‐based erosion evaluations in Ethiopian watersheds, which identified analogous spatial patterns and magnitudes in contexts of intensive land use and insufficient soil conservation [23, 67]. The watershed′s average annual soil loss rate was 44 t ha^−1^ yr^−1^, which is higher than the ~18 t ha^−1^ yr^−1^ rate that is acceptable for Ethiopian highland soils [49]. These high values indicate an unsustainable loss that surpasses the natural replenishment capacity, aligning with observed erosional accelerations subsequent to intensified land use [65, 67, 69]. About 65.2 million tons of soil are lost from the watershed every year. This shows how important it is to plan for soil and water conservation in Choke mountain watershed. As a result, almost half of the Choke Mountain watershed was above the limit for soil loss tolerance (Table 1).

**Table 1:** Annual soil loss class and risk levels in the Choke Mountain watershed.

Soil loss (t/ha/yr.)	Area (ha)	Percentage	Severity level	Assigned value	Risk level
< 5	304,972.55	15.25	Very slight	1	Very low
5–15	405,963.46	20.3	Slight	2	Low
15–30	270,975.61	13.55	Moderate	3	Medium
30–50	221,780.038	11.09	High	4	High
50–75	340,569.346	17.03	Very high	5	Very high
> 75	450,359.464	22.52	Severe	5	Very high

#### 3.1.2. Consistency and Validation of Model Estimates

The estimated average soil loss rates and their spatial distribution identified in this study align with field evidence and prior experimental results. Using rill and inter‐rill erosion measurements, Bewket and Sterk [70] found that some parts of the same and nearby watersheds lost between 18 and 79 t of soil per hectare per year. In the same way, a 5‐year study in the Anjeni experimental microwatershed in the study area found that traditional farming methods caused soil loss from cultivated land at rates between 17 and 176 t ha^−1^ yr^−1^ [71]. Belayneh et al. [23] found that newly built bunds, older graded bund‐treated plots, and untreated plots in the Gumara subwatershed′s croplands lost an average of 23.5, 45.6, and 58.1 t ha^−1^ yr^−1^ of soil, respectively. Hurni [49] also shows that cultivated land in the Ethiopian highlands lost an average of 42 t ha^−1^ yr^−1^ of soil, taking into account sediment redeposition. Additional validation comprised targeted field checks, where the color‐printed soil erosion severity map made by the model was compared with real‐life conditions on the site, which further proved that the model was accurate.

The estimates of soil loss from this study are very similar to those from previous real‐world studies. For instance, Miheretu and Yimer [72] said that the Gelana subwatershed in North Wollo lost an average of 24.3 t of soil per hectare per year. Gashaw et al. [14] noted 23.7 t ha^−1^ yr^−1^ in the Geleda watershed, and Tiruneh and Ayalew [67] noted 24.9 t ha^−1^ yr^−1^ in the Enfraz watershed. Haregeweyn et al. [27] found that the average amount of soil lost in the upper Blue Nile basin was 27.5 t ha^−1^ yr^−1^. The same thing happened in the Jabi Tehinan district of the West Gojjam Zone, where Amsalu and Mengaw [73] found that the average soil loss was 30.6 t ha^−1^ yr^−1^. These studies together back up the soil loss numbers in this research as being accurate and reliable.

The findings of this study corroborate previous research. For example, Molla and Sisheber [28] found that the Koga watershed in the upper Blue Nile basin lost an average of 47.4 t of soil per hectare per year, and Zerihun et al. [74] found that the Dembecha District in West Gojjam lost an average of 49 t of soil per hectare per year. Conversely, numerous studies from various regions of Ethiopia have demonstrated significantly elevated soil loss values. According to Zeleke [75], the northwestern highlands lose an average of 243 t of soil per hectare per year. This is mostly because of steep slopes, not enough plants, and poor land management. In the Chemoga watershed, Bewket and Teferi [25] found that the average amount of soil lost was 93 t ha^−1^ yr^−1^. In the upper Blue Nile basin, Tamene et al. [76] found that the average amount of soil lost was 75 t ha^−1^ yr^−1^. These studies show that soil erosion is common in the Ethiopian highlands, but the rates that are reported are very different. These differences are probably because of differences in how the research was done and the main things that cause erosion, such as the type of rain, the type of soil, the type of terrain, the type of land use, and the type of management practices.

##### 3.1.2.1. Soil Compaction.

The bulk density values in the Choke Mountain watershed ranged from 1 to 1.55 g cm^−3^. Table 2a shows that 80.24% of the area had a bulk density of 1–1.25 g cm^–3^, which means the soil was moderately compacted (Figure 4). This means that most of the watershed has medium soil compaction, which is probably because heavy farm equipment is not used much in the area. Highland areas with more rain and cooler temperatures, which are typical of these areas, had lower compaction levels. These areas had cooler temperatures and thick vegetation cover. Overall, the results show that soil compaction is not a major reason for land degradation in the Choke Mountain watershed.

**Table 2:** Statistics for physical land degradation indicators.

Factor	Classes	Area (Ha)	Percentage	Assigned value	Degradation level
a. Soil bulk density (g/cm^3^)	< 1	9998.10	0.50	1	Very low
	1–1.25	40,392.32	2.02	2	Low
	1.25–1.55	1,604,495.12	80.24	3	Moderate
	1.55–1.77	344,934.46	17.25	4	High
b. Level of drainage	Poor	0.06	0.00	1	Very low
	Imperfect	3502.61	0.18	2	Low
	Moderate	138,876.79	6.96	3	Moderate
	Well	370,223.85	18.56	4	High
	Somewhat excessive	1,433,703.58	71.88	5	Very high
	Excessive	0.06	0.00	6	Very high
c. Soil depth class (cm)	25–30	722.96	0.04	5	Very high
	50–100	329,127.34	16.50	4	High
	100–150	872,992.35	43.77	3	Moderate
	150–200	791,777.82	39.70	2	Low
	> 200	722.96	0.04	1	Very low

##### 3.1.2.2. Soil Drainage.

The spatial assessment of soil drainage indicates that approximately 74.4% of the watershed possesses well‐drained soils (Table 2b), as illustrated in Figure S6. The drainage map that was made is very similar to what people in the Amhara Regional State have seen and is in line with the drainage maps made by the [77].

##### 3.1.2.3. Soil Depth.

The watershed′s soil depth ranges from 25 to 175 cm (Figure S6). Table 2c shows that very deep soils (> 150 cm) cover about 43.77% of the area, which means that the land is not very degraded. On the other hand, about 39.7% of the watershed has shallow soils (25–30 cm), which shows a lot of damage. The downstream areas, where the soil is very shallow, are more prone to erosion, whereas the midland areas, where the soil is usually deeper, are less likely to be damaged.

### 3.2. The State of Physical Land Degradation

**Figure 5:**
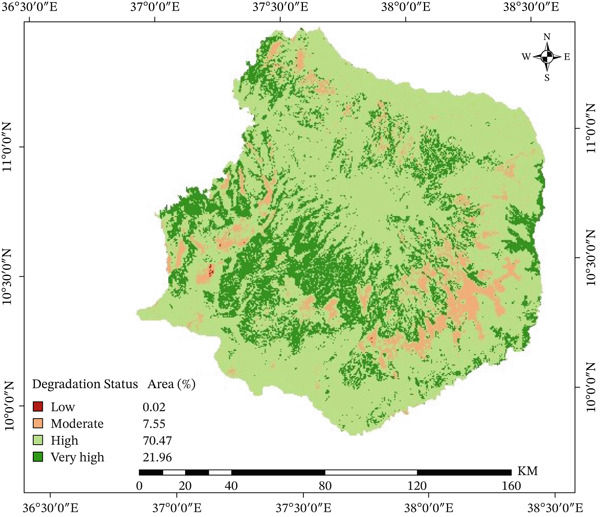
State of physical land degradation in the Choke Mountain watershed.

We used a pairwise comparison method to give four indicators that affect physical land degradation, soil erosion, soil compaction, soil drainage, and soil depth, relative weights. Each subclass was given a weight based on how much it affected degradation, and the comparison matrix showed a consistency ratio of 0.01, which shows that it was very reliable. The overlay analysis revealed that 70.7% of the watershed is in a state of moderate physical degradation. The pairwise comparison (Table 3) showed that soil drainage was the most important factor, followed by soil depth, soil erosion, and soil compaction (Figure 5). This means that most of the watershed′s physical characteristics are only slightly degraded [65].

**Table 3:** Matrix for pairwise comparisons of markers of physical land deterioration.

Criteria	Soil drainage	Soil depth	Soil erosion	Soil compaction	Criteria weighting
Soil drainage	1	2	3	5	40
Soil depth	0.5	1	2	3	30
Soil erosion	0.33	0.50	1	3	20
Soil compaction	0.2	0.33	0.33	1	10

Shallow soils and areas with little plant cover were often found together, suggesting that the soil was eroding quickly. Data collected from nearby farmers through formal and informal conversations confirmed these findings, showing that the soil was not penetrating well, the soil depth was decreasing, and erosion rates were rising. Farmers also said that soil compaction is getting worse because it makes it harder for water to get through and makes erosion worse. Surface runoff and ongoing farming, especially on steep terrain and cultivated fields, make the loss of soil depth worse. In general, the results show that the Choke Mountain watershed is getting worse because of physical land degradation (Figure 5). This is in line with what Haregeweyn et al. [27] found, which was that water‐driven erosion is a major cause of land degradation in the upper Blue Nile basin.

### 3.3. Biological Land Degradation Indicators

#### 3.3.1. Vegetation Cover

**Figure 6:**
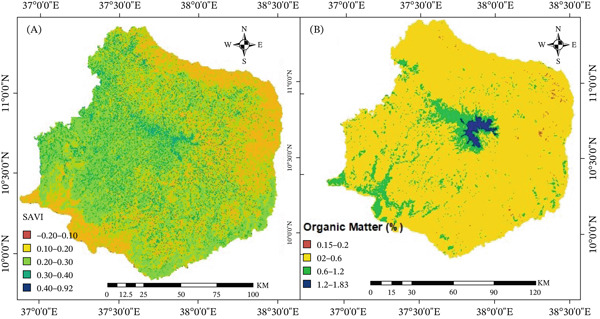
(A) Soil adjusted vegetation index and (B) soil organic matter (b) in Choke Mountain.

The Choke Mountain watershed′s vegetation index values ranged from −0.2 to 0.92 (Figure 6). Table 4 shows that 35.52% of the watershed had low vegetation cover and 41.47% had moderate vegetation cover, indicating moderate and high levels of land degradation, respectively. Furthermore, according to vegetation‐related indicators, almost one‐third of the watershed was classified as having high‐to‐very high degradation (Table 4; Figure 6). Principally in regions with little vegetation and grassland coverage, the lowland regions experienced more severe degradation than the highlands, highlighting the critical role that vegetation plays in maintaining ecological stability. This result is consistent with the study conducted by Ewunetu [65], which found that areas covered by vegetation and grass are less vulnerable to soil loss.

**Table 4:** Mean soil‐adjusted vegetation index (SAVI) statistics.

SAVI classes	Area (ha)	Area (%)	Cover status	Assigned values	Degradation level
< 0.1	85,992.26	4.30	Very poor	5	Very high
0.1–0.2	710,336.07	35.52	Poor	4	High
0.2–0.3	829,325.35	41.47	Moderate	3	Moderate
0.3–0.4	327,170.55	16.36	High	2	Low
> 0.4	46,995.77	2.35	Very high	1	Very low

#### 3.3.2. Status of SOM

The SOM status in the Choke Mountain watershed varied from 0.15% to 1.87% (see Figure 6). According to Table 5, around 89.02% of the watershed showed SOM levels ranging from 0.2% to 0.6%, which is considered low and indicates a significant decline in soil quality. Low levels of SOM adversely affect soil fertility and the functioning of ecosystems because they are closely linked to increased soil erosion and reduced microbial activity. Conversely, higher SOM levels signify soils enriched with humus and organic remains from decomposed plant and animal materials. The generally low SOM levels in the watershed are likely a result of ongoing agricultural practices and the removal of crop residues for uses such as household waste, animal feed, and fuel, which negatively influences soil productivity.

**Table 5:** Levels of soil organic matter of topsoil in the Choke Mountain watershed.

Category (%)	Area (Ha)	Percentage (%)	Level of SOM	Severity level	Assign value
0.15–0.2	5199.532	0.26	Very low	Very high	5
0.2–0.6	1,780,239.764	89.02	Low	High	4
0.6–1.2	190,182.882	9.51	Medium	Moderate	3
1.2–1.86	23,997.84	1.20	High	Low	2

### 3.4. The Status of Biological Land Degradation

A pairwise comparison matrix was used to compare the relative importance of two main indicators of biological land degradation: vegetation cover and SOM. This was done in the same way as for the physical degradation indicators. The amount of damage each subclass caused to the overall degradation was used to give weights. According to Table 6, SOM had a bigger effect on the biological degradation of land in the Choke Mountain watershed than vegetation cover did. The pairwise comparison matrix showed a consistency ratio of 0.01, which means that the weights are very reliable and correct.

**Table 6:** A comparison matrix of the biophysical indices of land degradation.

Criteria	Organic matter	Vegetation cover	Criteria weighting
Organic matter	1	3	70
Vegetation cover	0.33	1	30

**Figure 7:**
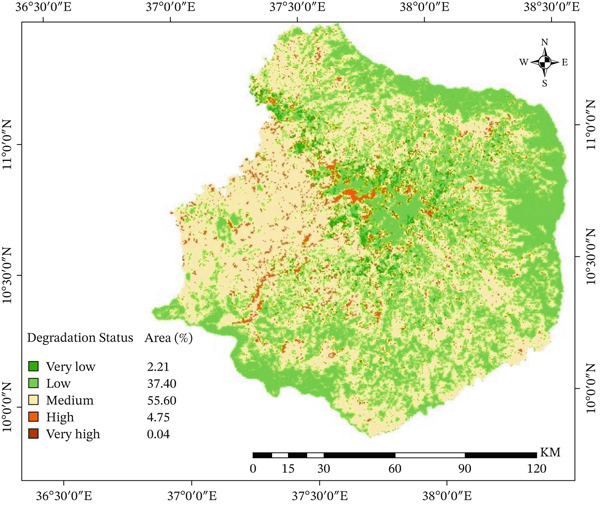
Biological land degradation status in the Choke Mountain watershed.

Figure 7 shows a weighted overlay analysis that shows that 37.4% and 55.5% of the watershed are affected by biological degradation that is low to moderate. This means that almost half of the watershed is experiencing biological decline, mostly because there is less vegetation cover and less SOM. The results have major effects: Less SOM not only makes the soil less fertile but also makes it harder for plants to grow, which makes the ecosystem more vulnerable. In the same way, having little plant cover on the ground makes it less safe, which causes erosion and loss of nutrients. People who use the land in the area, both formally and informally, confirmed these findings. They showed that using too many natural resources has slowly hurt local plants and animals and that losing nutrients from soil due to water erosion is still a big problem. These results align with previous research conducted in northeastern Ethiopia, including Ewunetu and Kerbsh [5], which demonstrated that biological land degradation is widespread and ongoing, substantially impacting agricultural productivity and ecosystem stability.

### 3.5. The State of Chemical Land Degradation

**Figure 8:**
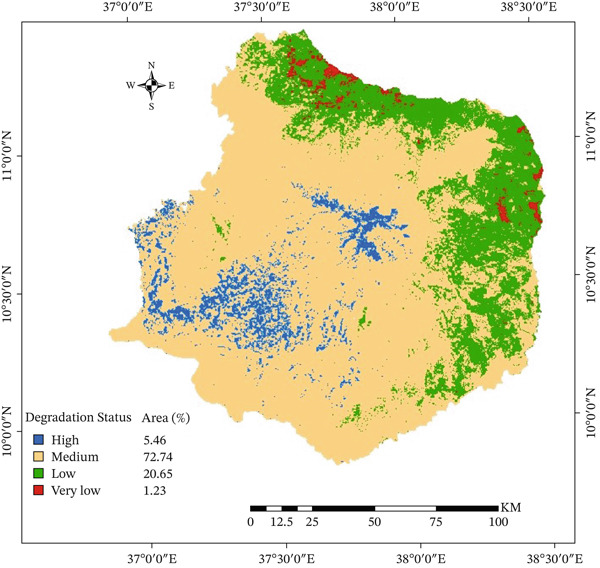
Soil acidity statuses in the Choke Mountain.

The Choke Mountain watershed had chemical land degradation that ranged from 5 to 7.8, as measured by soil acidity (pH) (Figure 8, Table 7). About 4% of the watershed had very acidic soils, with pH values below 5.5. The Choke Mountain reserved areas, which have a wet climate, lots of water, and thick natural forest and Afroalpine grass cover, had the highest levels of soil acidity. In these areas, heavy rain and wet conditions cause important cations like calcium and magnesium to leach out of the soil. Aluminum takes its place, which makes the soil more acidic [78]. The breakdown of organic matter also releases hydrogen ions, which makes the soil even more acidic [79].

**Table 7:** The status of soil acidity in Choke Mountain.

Soil pH	Area (Ha)	Percentage (%)	Level	Assigned value
5–5.5	78,984.99	3.95	High	4
5.5–6.7	779,651.85	38.99	Medium	3
6.7–7.3	1,116,387.86	55.83	Low	2
7.3–7.8	24,595.33	1.23	Very low	1

About 37.4% of the watershed had soil pH values between 5.5 and 6.7. This indicates that chemicals were breaking down quickly (Table 7). These moderately acidic soils were mostly located in the highland and midland areas. Here, eucalyptus plantations, continuous farming, and repeated use of chemical fertilizers are common (Figure 8). Using acid‐forming fertilizers like ammonium phosphate (DAP) and urea over and over again on different crops, along with taking away crop residues for fuel and livestock feed, has made the soil much more acidic [79]. Eucalyptus plantations were also found to be major causes of acidification because they take up a lot of nutrients [78]. These findings show that human activities mainly cause chemical land degradation in the Choke Mountain watershed. Excessive fertilizer use, continuous cultivation, and certain land‐use practices harm soil fertility and long‐term agricultural productivity. To protect the health of the ecosystem and maintain agricultural productivity in the watershed, it is essential to address soil acidity with integrated soil fertility management and conservation strategies.

Most of the watershed (55.6%) had soils with pH between 6.7 and 7.3, indicating they were neutral. Only 1.23% of the area was alkaline, with pH levels between 7.3 and 7.8 (Table 7; Figure 8). The lowland and driest parts of the watershed had most of the soils that were not very acidic. These findings indicate that approximately 50% of the Choke Mountain watershed is susceptible to chemical land degradation, despite the absence of significantly acidic soils. Key informants said that lime use on croplands has increased over the last 15 years because the soil has become more acidic, especially in the middle and upper parts of the watershed, where crops are grown most. However, lime use is still limited because there is not enough of it, which makes it harder to reclaim acidic soils [65]. Overall, the results show that chemical degradation is becoming more of a problem in areas with a lot of farming, and that making lime more available and getting more people to use it is important for reducing soil acidification and keeping crop yields high.

### 3.6. Integrated Land Degradation Vulnerability in Choke Mountain

**Figure 9:**
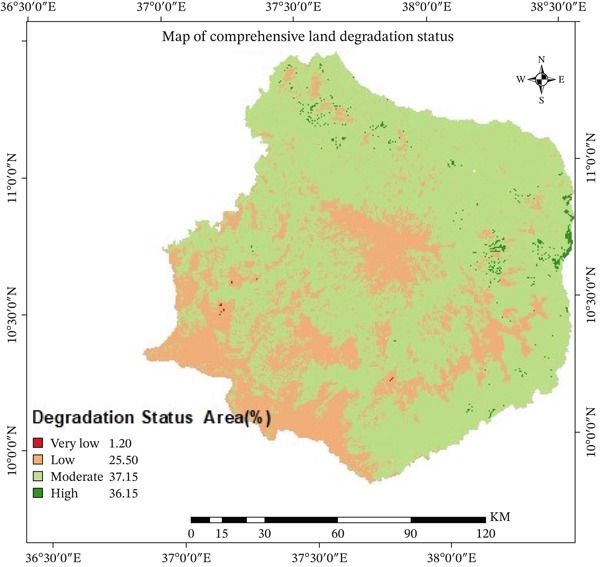
Comprehensive land degradation map of the north Gojjam subbasin.

The final comprehensive land degradation index for the Choke Mountain watershed was created by combining biological, physical, and chemical indicators. The cell size of all parameter raster layers was changed to 30 × 30 m, and the projection was changed to UTM Zone 37°N, WGS 1984 datum. The land degradation vulnerable area map was then divided into five groups: (1) very low, (2) low, (3) moderate, (4) high, and (5) very high level (Figure 9). The pairwise comparison matrix (Table 8) shows that biological indicators had the biggest impact on land degradation as a whole, followed by physical and chemical indicators. The weighted comparison produced a consistency ratio of 0.08, signifying satisfactory reliability (< 0.10).

**Table 8:** Pairwise comparison matrix of land degradation status in the north Gojjam subbasin.

Criteria	Biophysical degradation	Physical degradation	Chemical degradation	Criteria weighting
Biophysical degradation	1	3	7	68
Physical degradation	0.33	1	2	22
Chemical degradation	0.14	0.5	1	10

According to Figure 9, about 25.5% of the watershed is in a state of low degradation, 37.15% is in a state of moderate degradation, and 36.5% is in a state of high degradation. The most damaged areas are mostly in the lower parts of the watershed. This is because of a combination of steep slopes, constant farming, rough terrain, a growing population, bad land management, and unpredictable rainfall. Most of the moderately degraded zones are in the middle of the elevation range, where the land is relatively flat and less likely to erode. On the other hand, the very top of the watershed has areas with very little degradation, where natural forests and Afroalpine plants are the most common.

People who use the land in the area confirmed these findings by saying that soil erosion, low vegetation cover, less organic matter in the soil, and high acidity in the soil were the main causes of land degradation. Communities also stressed that climate change, bad farming practices, bad grazing management, and soil quality that is getting worse have all made soil erosion and land degradation worse. In total, more than 71% of the watershed is moderately to highly degraded. This shows that land degradation is a major environmental and social problem in the Choke Mountain area. These findings are consistent with earlier research in the upper Tekeze basin [5], the Omo‐Gibe River basin [29], and the semiarid watershed of Rajasthan, India [31], corroborating the prevalence of land degradation in similar highland and semiarid ecosystems. The findings of this study correspond with those of national‐level research [7, 9], indicating significant land degradation in the northeastern, eastern, and southeastern regions of the country, where rain‐fed agriculture predominates, and soil conservation measures are minimally applied. Moreover, the findings of this study align with those of Ewunetu [65] and, which found that land degradation is a severe problem in the north Gojjam subbasin.

The fact that so much land is moderately to severely degraded shows how important it is to have integrated land management plans that include things like protecting soil and water, planting trees, better grazing practices, and sustainable agricultural intensification. If these drivers are not dealt with, soil fertility may continue to decline, agricultural productivity may drop, and ecosystem services may be harmed, which would have serious effects on the local communities′ economies and societies.

### 3.7. Conclusions and Policy Implications

This study used RUSLE MCA and HAP in a GIS environment to map and measure land degradation in the Choke Mountain watershed in Ethiopia. It did this by combining different physical, biological, and chemical indicators of degradation. The study found that the Choke Mountain watershed lost an average of about 44 t ha^−1^ yr^−1^, and 45.3% of the watershed was at high to very high risk. Biological degradation was the worst, while chemical and physical degradation were not as bad. In general, more than 71% of the watershed is in a state of moderate to high degradation, which shows how important it is to restore the land and manage it in a way that is good for the environment.

Major policy implications include:

• Improving soil fertility: Use lime and organic materials like compost, manure, and mulching to fix soil acidity and nutrient loss.
• Preventing erosion: Encouraging agroforestry systems and multipurpose perennial crops to reduce topsoil erosion and make slopes more stable.
• Strengthening collaboration among stakeholders: Farmers, local communities, government agencies, and development partners working together to carry out effective land rehabilitation measures

These findings indicate that the amalgamation of GIS, remote sensing, and MCA is exceptionally efficient for pinpointing priority intervention zones. To stop land degradation and help people in highland areas make a living in a way that is good for the environment, it is important to keep an eye on things and change management plans based on the local biophysical and socioeconomic conditions.

## Author Contributions

Alelgn Ewunetu was the only person who did all the research and wrote the paper.

## Funding

No funding was received for this manuscript.

## Ethics Statement

All procedures in this study were conducted in compliance with applicable guidelines and regulations. Ethical approval was granted by the Department of Geography and Environmental Studies at Woldia University, and an official confirmation letter is provided as Supporting Information. Formal authorization to carry out data collection was obtained from the department. Participants provided informed consent before their involvement. Because the study was conducted after the conflict between the Federal Government and local rebel groups, many participants expressed concerns about possible political consequences and were unwilling to provide written consent. As a result, verbal consent was secured after clearly explaining the study′s objectives, data usage, and participants′ right to withdraw at any time without penalty. Individuals under 18 years of age were not included in the study. All collected data were treated with strict confidentiality. Personal identifiers were recorded solely for verification purposes and were protected until transcription and analysis were finalized. Consent for publication was also obtained verbally, and anonymity was ensured by reporting responses in aggregated form.

## Conflicts of Interest

The author declares no conflicts of interest.

## Supporting information


**Supporting Information** Additional supporting information can be found online in the Supporting Information section. Table S1: C‐factor for different LULC types. Table S2: P‐factor of conservation practices [25]. Table S3: Soil drainage classes [36]. Table S4: Soil compaction level (USDA 2017). Table S5: Soil depth classes (USDA 2017). Table S6: Classes of soil organic matter in soil (Eastman 2012). Table S7: Category of soil acidity level based on pH value [64]. Figure S1: Flow chart showing the methodology for soil loss estimation. Figure S2: Rainfall erosivity (right) and soil erodibility (left) in Choke Mountain. Figure S3: Elevation (right) and LS‐factor (left) maps of the Choke Mountain. Figure S4: Cover factor values (right) and LULC types (left) in Choke Mountain. Figure S5: Slope class (right) and management values (left) in the Choke Mountain. Figure S6: Soil bulk depth (right) and soil drainage (left) in Choke Mountain watershed.

## Data Availability

The data used in this study are available from the corresponding author on reasonable request.
