# Supplementary material for: Spatial Assessment of Land Degradation Risk in the Choke Mountain Watershed
Source: ScientificWorldJournal. 2026 Apr 25;2026:6657991. doi: 10.1155/tswj/6657991 (PMC13110348; doi:10.1155/tswj/6657991)
Supplement: Supplementary file 1 — Supporting Information Additional supporting information can be found online in the Supporting Information section. Table S1: C‐factor for different LULC types. Table S2: P‐factor of conservation practices [25]. Table S3: Soil drainage classes [36]. Table S4: Soil compaction level (USDA 2017). Table S5: Soil depth classes (USDA 2017). Table S6: Classes of soil organic matter in soil (Eastman 2012). Table S7: Category of soil acidity level based on pH value [64]. Figure S1: Flow chart showing the methodology for soil loss estimation. Figure S2: Rainfall erosivity (right) and soil erodibility (left) in Choke Mountain. Figure S3: Elevation (right) and LS‐factor (left) maps of the Choke Mountain. Figure S4: Cover factor values (right) and LULC types (left) in Choke Mountain. Figure S5: Slope class (right) and management values (left) in the Choke Mountain. Figure S6: Soil bulk depth (right) and soil drainage (left) in Choke Mountain watershed. [file TSWJ-2026-6657991-s001.pdf]

Appendexes

Table S1: C-factor for different LULC types

LULC	C-value	Sources
Dense Forest	0.01	(Bewket & Teferi, 2009; Ewunetu et al., 2021a)
Shrub and bush	0.20	(Eshetu & Abegaz, 2024; Ewunetu et al., 2021a)
Grazing land	0.05	(Hurni,1985)
Cultivatted land	0.15	(Tiruneh & Ayalew, 2015)
Barren land	0.60	(Bewket & Teferi, 2009; Ewunetu et al., 2021a)
Waterbodies	0.00	(Ewunetu et al., 2021a)

Table S2: P-Factor of conservation practices (Bewket & Teferi, 2009)

Land use type	Slope (%)	P-value
Agricultural land	0-5	0.1
	5-10	0.12
	10-20	0.14
	20-30	0.19
	30-50	0.25
	50-100	0.33
Non-agricultural land	0-100	1

Table S3: Soil drainage classes (Hengl et al., 2017)

Drainage class	Level drainage	Level description
1	Very poor	Drained Excessively
2	Poor	Drained Somewhat Excessively
3	Imperfect	Well Drained
4	Moderate	Well-drained moderately
5	Well	Drained Somewhat Poorly
6	Somewhat excessive	Poorly Drained
7	Excessive	Drained Very Poorly

Table S4: Soil compaction level (USDA, 2017)

Bulk density class	Compaction Status
< 1 g/cm^3	Low compacted soil
1-1.25 g/cm^3	Medium compacted soil
1.25-1.55 g/cm^3	Highly compacted soil
> 1.55 g/cm^3	Very high compacted soil

Table S5: Soil depth classes (USDA,2017)

Soil depth class	Degradation level	Level Description
Less than 30cm	Very high	Soil has a very shallow depth
From 30 to 50cm	High	Soil has a shallow depth
From 50 to 100 cm	Moderate	Soil has moderately deepth
From 100 to 150 cm	Low	Soil has a high depth
Greater 150 cm	Very Low	Soil has a very deep depth

Table S6: Classes of soil organic matter in soil (Eastman, 2012)

Category in %	Description
< 0.2	Very poor soil organic matter content in the soil
0.2-0.6	Low level of organic materials in the soil
0.6-1.2	Medium soil organic matter content in soils
1.2-2.0	High level amount of soil organic matter
>2.0	An extremely high amount of organic materials in the soil

Table S7: Category of soil acidity level based on pH value (Nachtergaele et al., 2008).

PH value	Description
< 5.5	Very acidic soils
5.5 - 6.7	Soils that are somewhat acidic
6.7-7.3	Soils that are neutral
7.3-8.0	Soils that are somewhat alkaline
> 8.0	Extremely alkaline soils

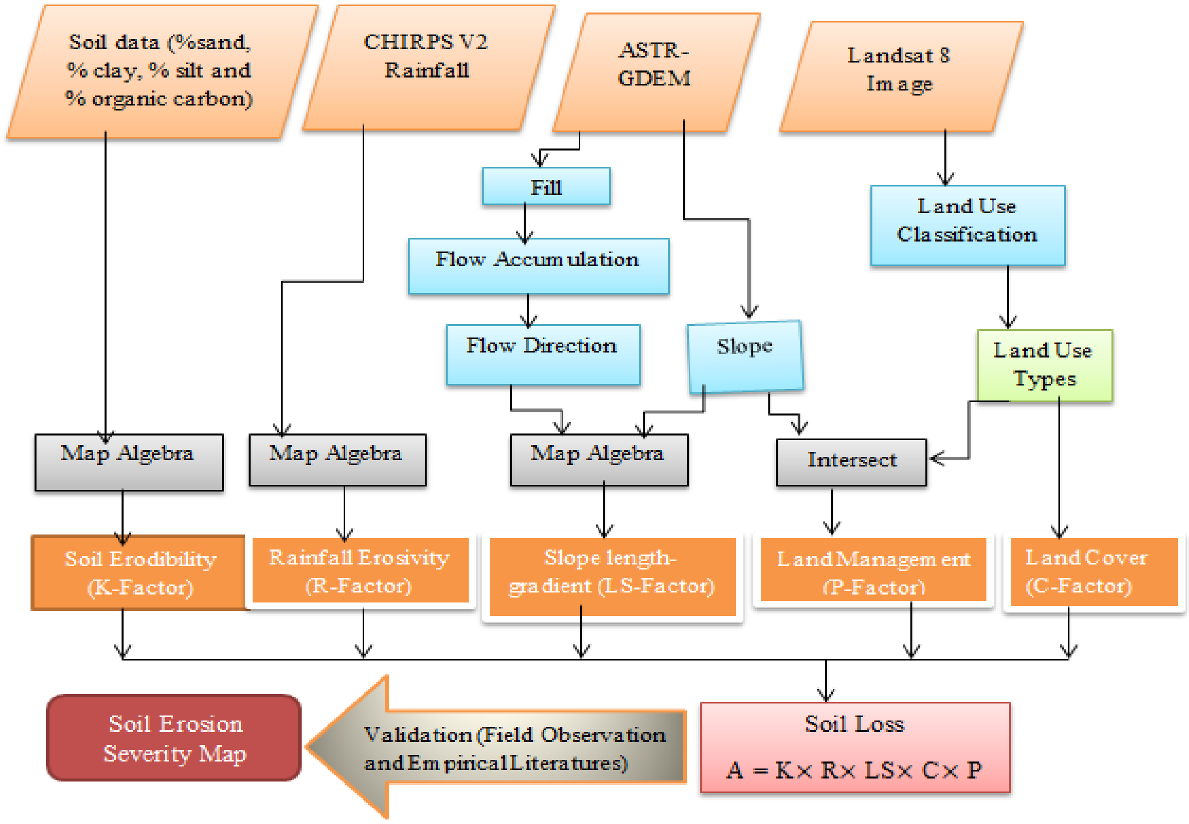

Fig. S1: Flow chart showing the methodology for soil loss estimation.

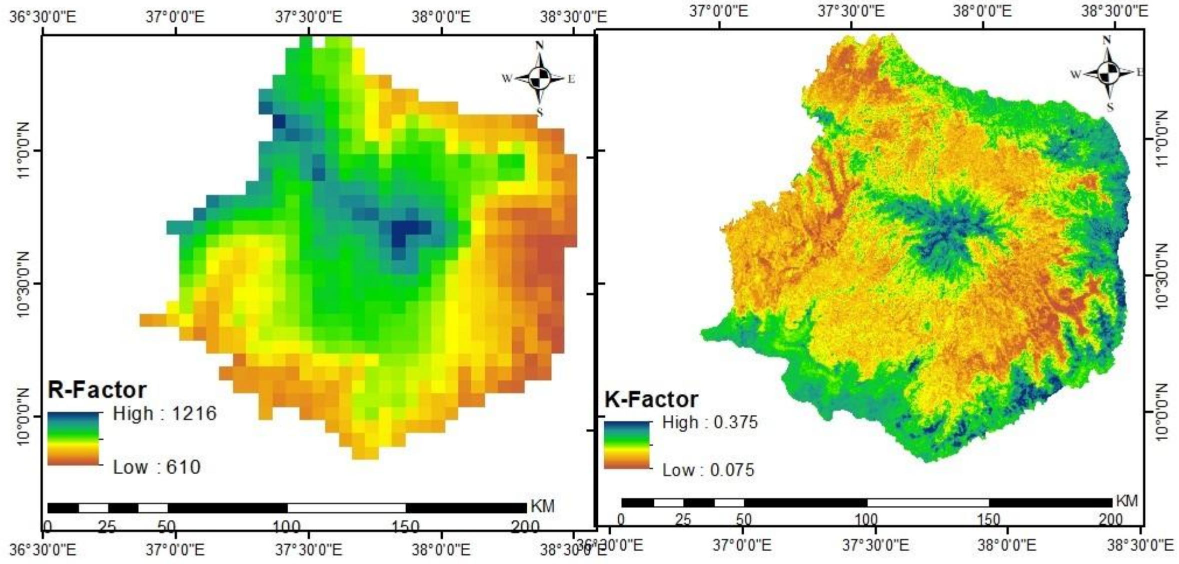

Fig. S2: Rainfall erosivity (right) and soil erodiabilty (left) in Choke Mountain.

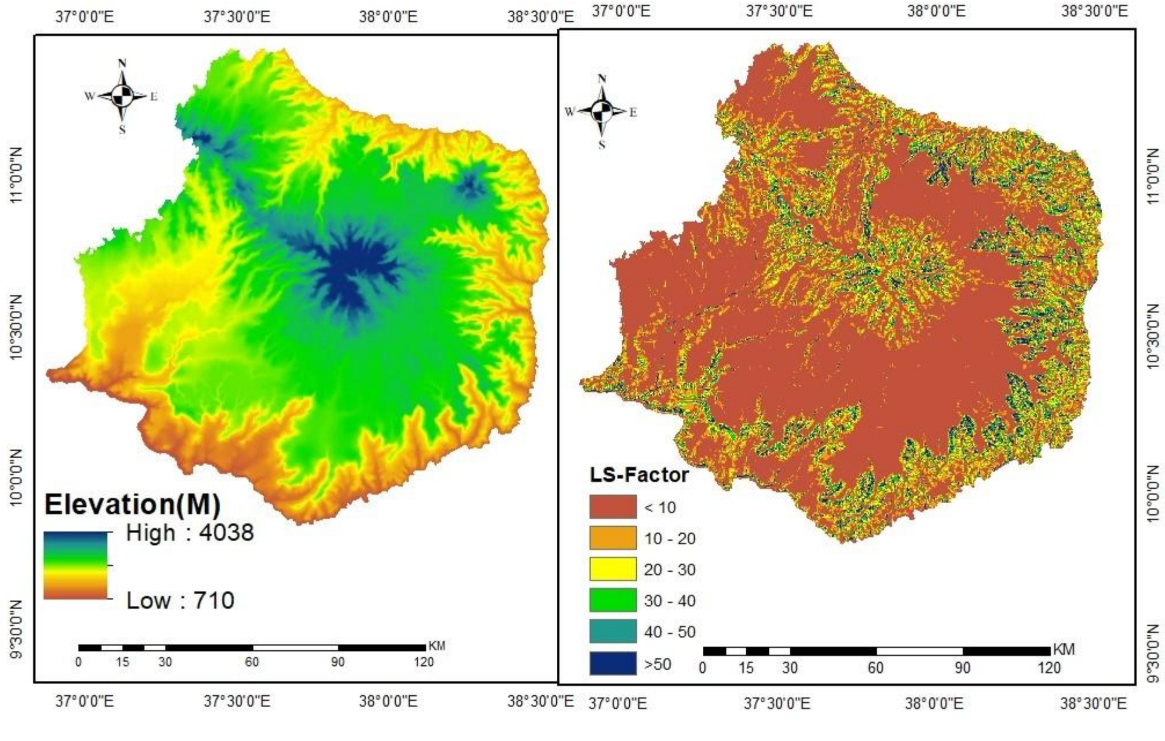

Fig. S3: Elevation (right) and LS-factor (left) maps of the Choke Mountain

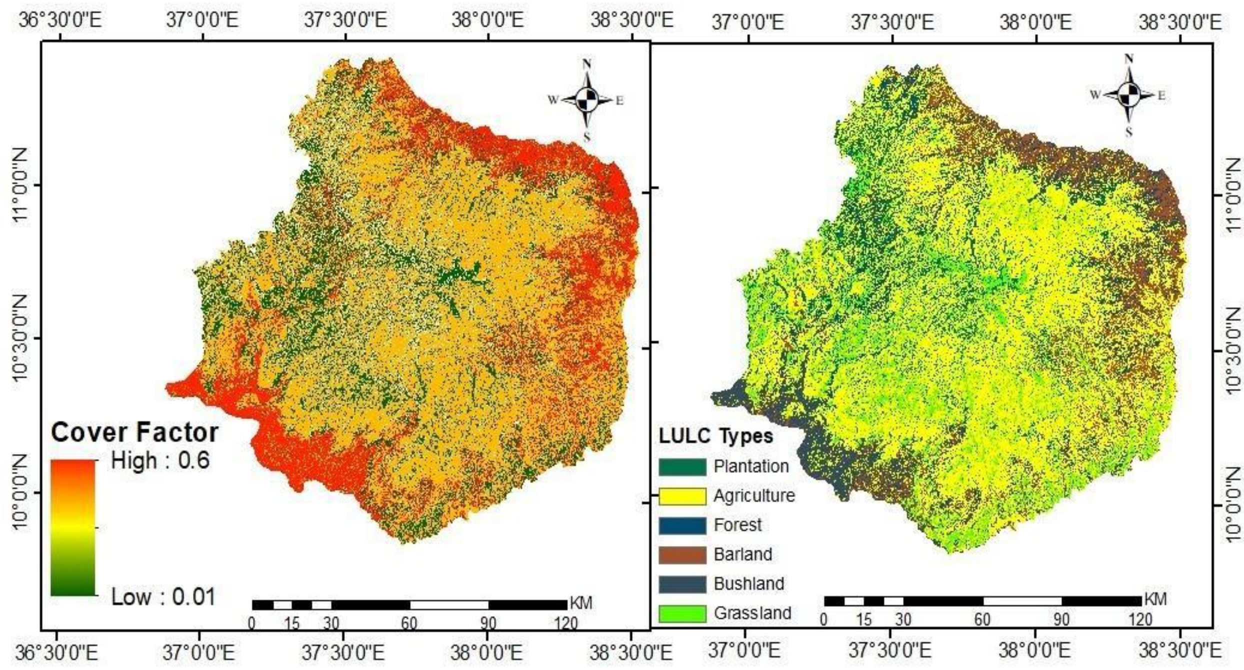

Fig. S4: Cover factor values (right) and LULC types (left) in Choke Mountain

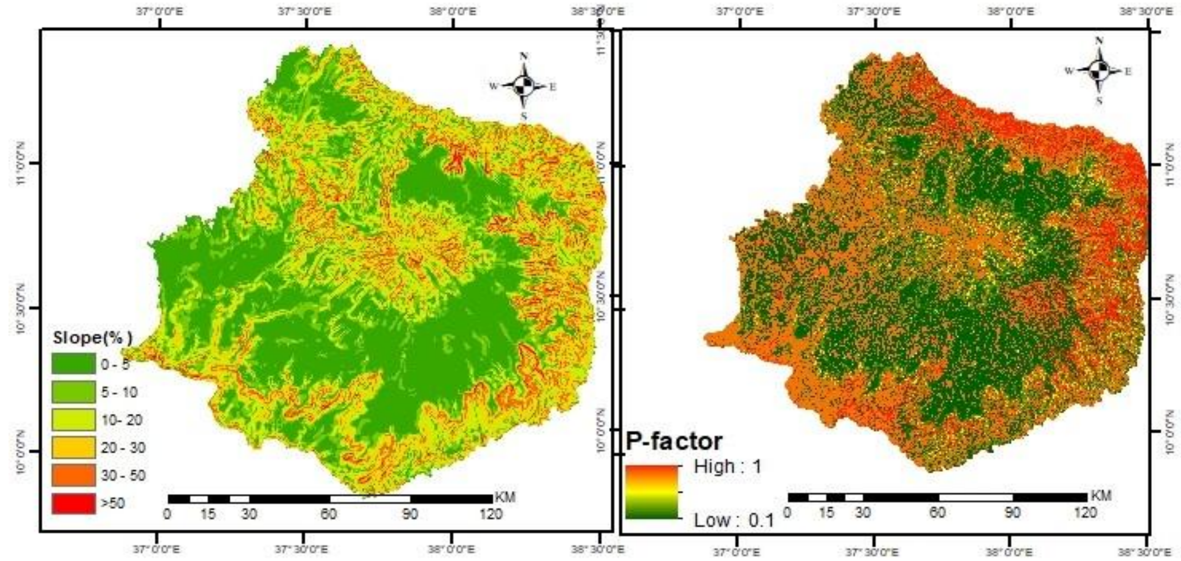

Fig. S5: Slope class (right) and management values (left) in the Choke Mountain.

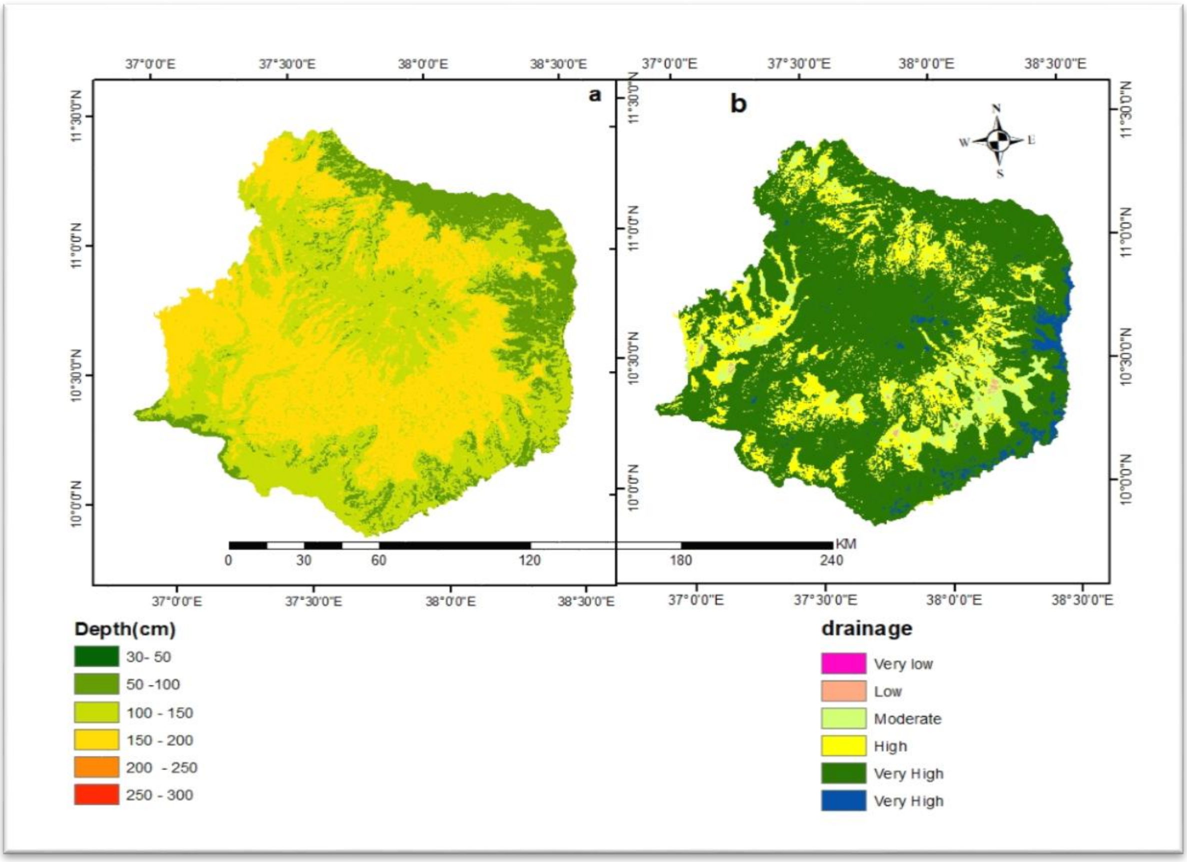

Fig. S6: Soil bulk depth (right) and soil drainage (left) in Choke Mountain watershed
